# Early Post-Stroke Infections Are Associated with an Impaired Function of Neutrophil Granulocytes

**DOI:** 10.3390/jcm9030872

**Published:** 2020-03-23

**Authors:** Till van Gemmeren, Ramona Schuppner, Gerrit M. Grosse, Jessica Fering, Maria M. Gabriel, René Huber, Hans Worthmann, Ralf Lichtinghagen, Karin Weissenborn

**Affiliations:** 1 Department of Neurology, Hannover Medical School, 30625 Hannover, Germany; T.vanGemmeren@gmx.de (T.v.G.); Grosse.Gerrit@mh-hannover.de (G.M.G.); Jessica.Fering@web.de (J.F.); Gabriel.Maria@mh-hannover.de (M.M.G.); Worthmann.Hans@mh-hannover.de (H.W.); Weissenborn.Karin@mh-hannover.de (K.W.); 2 Institute of Clinical Chemistry, Hannover Medical School, 30625 Hannover, Germany; Huber.Rene@mh-hannover.de (R.H.); Lichtinghagen.Ralf@mh-hannover.de (R.L.)

**Keywords:** stroke, inflammation, neutrophils, immunodepression, oxidative burst

## Abstract

To investigate whether neutrophil granulocytes’ function relates to post-stroke infections and clinical outcome after stroke, we prospectively recruited 95 patients after ischemic stroke and tested them for their microbiocidal neutrophil functions in this exploratory study. Additionally, 24 age-adjusted controls were examined regarding neutrophil function. Phagocytic capacity and the ability of the neutrophil granulocytes to produce reactive oxygen species (ROS) as well as CD11b and CD16 receptor expression profile were measured by flow cytometry at days 1, 3, 7, and 90 after symptom onset. Primary outcome was the development of an infection within the first week after stroke. Results of neutrophil functional measurements were compared between patients with and without infection as well as between all stroke patients and controls. Further risk factors for the development of infections were summarized in an infection-risk score for the purpose of multivariate statistical analysis. The ROS production in neutrophils after stimulation with formyl-methionyl-leucyl-phenylalanine (fMLP) was reduced at baseline in patients with post-stroke infections compared to those without (*p* = 0.013). This difference proved to be independent from the infection-risk score in the binary logistic regression (*p* = 0.011). Phagocytosis and oxidative bursts were not significantly reduced in the whole stroke patient group compared to controls. Dysfunction of neutrophil granulocytes seems to play a significant role in the development of post-stroke infections. Further studies are warranted to investigate neutrophil granulocytes´ function as a potential biomarker of post-stroke infections.

## 1. Introduction

Infections—especially respiratory or urinary tract infections—are common complications after acute ischemic stroke affecting up to 30% of the patients [1,2]. The high infection rates among stroke patients have been attributed to a state of immunodepression due to the cerebral damage. Cellular immunity is impaired as monocytes are partly deactivated. This is represented by lowered HLA-DR/MHC class II expression, as well as reduced tumor necrosis factor α (TNF-α) production and reduced oxidative burst activity [3,4,5]. T-lymphocyte counts are reduced and show altered functional status with lower Interferon-γ production in Th1-cells of mice and humans [3,6]. Until now, there are only few and small studies dealing with the microbiocidal function of circulating neutrophil granulocytes after stroke: While ROS production is necessary for a successful defense against bacteria, it can also reinforce inflammatory post-ischemic damage and might therefore contribute to an unfavorable functional outcome after stroke [7,8]. An impaired production of oxidative bursts was shown in patients with hemorrhagic stroke and ischemic stroke compared to healthy controls [5,9]. In patients with ischemic stroke, a reduced amount of intracellular myeloperoxidase (MPO), a key enzyme of neutrophil defense mechanisms, was found [10]. 

The aim of this prospective, non-interventional, cohort study was to investigate whether neutrophil granulocytes’ function relates to post-stroke infections and clinical outcome 90 days after stroke. We hypothesized that patients with post-stroke infections show an impairment of neutrophil function at baseline and worse clinical outcome after 90 days compared to patients without post-stroke infections. 

## 2. Experimental Section 

### 2.1. Patients and Controls

One-hundred-fifty-two patients with acute focal neurological deficits were screened for study eligibility in the Department of Neurology at Hannover Medical School, Germany, between 2015 and 2017. The study was approved by the ethics committee of Hannover Medical School and was performed in accordance with institutional guidelines. All patients, their legal representatives, or close relatives gave written informed consent. Inclusion criteria were acute ischemic stroke with symptom onset <24 h prior to blood sampling and age >18 years. Exclusion criteria were clinically apparent infections on admission, fever, C-reactive protein (CRP) >30 mg/L, stroke within three months prior to admission, modified Rankin Scale (mRS) >3 prior to admission, known immunodeficiency, malignoma, and hemodialysis. Demographical and clinical data including cardiovascular risk factors (arterial hypertension, diabetes mellitus, hyperlipidemia, renal dysfunction, obesity, and smoking status) and stroke etiology, classified according to Trial of Org 10172 in Acute Stroke Treatment (TOAST) criteria, were obtained for all patients [11]. Obesity was defined as body mass index (BMI) ≥30 kg/m^2^, and renal dysfunction as an estimated glomerular filtration rate (eGFR) <60 mL/min/1.73 m^2^. Twenty-four controls adjusted for age where recruited among visitors of the Neurological Department at Hannover Medical School. Exclusion criteria were the same as mentioned above. All controls, but two, had medications and preexisting medical conditions but no acute disease (see Table 1). Neurological symptoms were evaluated using the National Institutes of Health Stroke Scale (NIHSS). Patients were screened for infections as part of the daily clinical routine and by appropriate diagnostics if applicable (e.g., chest X-ray, urine examination). Diagnosis of infection was performed in consensus by two experienced physicians, who were blinded for neutrophil granulocyte function, and was based on the recommendations from the Pneumonia in Stroke Consensus Group and the Centers for disease control and prevention (CDC)/ National Healthcare Safety Network (NHSN) surveillance definition of healthcare-associated infection [12,13]. Clinical outcome after 90 days was evaluated using the modified Rankin Scale (mRS) ranging from 0 (normal) to 6 (death), and the Barthel Index (BI) [14,15]. Unfavorable outcome was defined as mRS > 3 and/or BI < 60 as proposed by Sulter et al. [16].

### 2.2. Blood Sampling

Patients’ blood samples were taken within 24 h after symptom onset (baseline) and after 3, 7, and 90 days. In the case of intravenous thrombolytic or endovascular treatment, blood was drawn before treatment. Laboratory workup regarding the measurement of neutrophil function started within four hours after blood sampling. S100B, as a marker of the extent of cerebral damage, was determined three days after symptom onset, since at that time point its levels are known to correlate well with the infarct volume [17]. Neutrophil–lymphocyte ratio (NLR) was calculated as a surrogate marker for the inflammatory reaction and the extent of systemic stress, potentially indicating higher risk of post-stroke infections, as described recently [18,19]. C-reactive protein (CRP) served as marker of inflammation.

### 2.3. Neutrophil Function

Reactive oxygen species (ROS) production and extent of phagocytosis by neutrophils was determined using the flow cytometry-based commercial test kits PHAGOBURST™ and PHAGOTEST™ (Glycotope Biotechnology) according to the manufacturer’s instructions with minor changes (see Appendix A). Gating was performed by selecting cells with the DNA content of a diploid cell in a histogram. Then, in the forward scatter/side scatter (FSC/SSC) scatterplot of selected cells, neutrophils were gated. After excluding doublets in a forward scatter width/forward scatter height (FSC-W/FSC-H) scatterplot, the fluorescence signal of rhodamine 123-positive cells was gated in a histogram. The negative control was used to exclude spontaneous burst activity as well as autofluorescence and normal mitochondrial ROS production. 

### 2.4. Neutrophil Phenotype 

To assess the receptor expression profile of neutrophils, fluorescence-labeled CD16 and CD11b antibodies (BD Biosciences) were used. One-hundred microliter samples of precooled whole blood underwent erythrocyte lysis with Red Blood Cell (RBC) Lysis Buffer (BioLegend) followed by centrifugation (5 min, 300 g, 20 °C) and elution with 1 mL of phosphate-buffered saline (PBS)/2% fetal calf serum (FCS). Afterward, samples were incubated with 1 µL of PE Cy™5 Mouse Anti-Human CD16 or 5 µL of APC-Cy™7 Mouse Anti-Human CD11b for 20 min on ice. Samples were then centrifuged, and cell pellets resuspended in 300 µL of PBS/2% FCS. Cytometric analysis and data evaluation were performed as described above. 

Neutrophil function at baseline, 3, 7, and 90 days after stroke was assessed for all patients until the planned number of patients without infections had been recruited. Afterward, the assessment at day 7 and 90 was omitted in patients, who did not develop an infection within the first seven days after stroke, since the main hypothesis was based on the baseline neutrophil function.

### 2.5. Statistical Analysis

IBM SPSS Statistics 24 was used for statistical analysis. Figures were created using GraphPad Prism 7. For metric variables, median and inter quartile range (IQR) were calculated. Non-metric variables are presented as total numbers and percent. In univariate analyses, Mann–Whitney U test was used for testing metric, not normally distributed variables, while non-metric univariate testing was done by chi-square test. In the multivariate analysis a binary logistic regression with backward elimination was performed. Variables were chosen as covariates at a significance level of at least 95% in univariate testing (*p* < 0.05) and by suspected influence on the development of infections. An infection-risk score was set up in order to summarize known risk factors for the development of infections. It includes the presence of reduced consciousness, dysphagia, a urinary catheter, invasive ventilation, a nasogastric feeding tube, and the NIHSS at baseline as well as S100B levels at day 3 (see Table S1).

### 2.6. Data Availability Statement

The data that support the findings of this study are available from the corresponding author upon reasonable request.

## 3. Results

### 3.1. Group Characteristics

Of 152 patients recruited in the emergency room, 57 had to be excluded since they did not fulfill the inclusion criteria or had exclusion criteria (see Table S2). Thus, finally, 95 patients were included in the study. Thirty-eight patients (40%) were female, the median age was 76 (IQR: 65–82) years. In 64 patients, the exact time of symptom onset was known. The median time interval from symptom onset to the first blood sampling was 7.46 (IQR: 1.85–18.42) h. Twenty-seven patients out of 95 (28%) developed infections within the first seven days after stroke (*n* = 8 urinary tract infections, *n* = 12 lower respiratory tract infections, *n* = 4 infections without focus, and one each with erysipelas, upper respiratory tract infection, and gastro-intestinal infection). Patients with infections had a more severe stroke (NIHSS at baseline and S100B at day 3; *p* < 0.001 and *p* < 0.001), had no microangiopathic strokes (*p* = 0.02), were more often obese (*p* = 0.015), showed higher NLR (*p* = 0.001), and scored higher in the infection-risk score (*p* < 0.001) than patients without infection (see Table 1). mRS and/or BI at day 90 could be obtained for 87 patients. Eight patients, of whom two had no 7 and 90 day cytometric measurement planned, were classified as complete loss to follow-up. Twenty-six of the 87 patients (30%) showed an unfavorable outcome (mRS > 3 and/or BI < 60) including 17 with infection (see Table 1). In comparison to stroke patients, controls had a lower occurrence of atrial fibrillation (*p* = 0.008) and arterial hypertension (*p* = 0.001). Additionally, baseline characteristics in controls did not differ significantly from those in stroke patients.

### 3.2. Oxidative Burst

#### 3.2.1. Burst Activity per Cell

**Figure 1:**
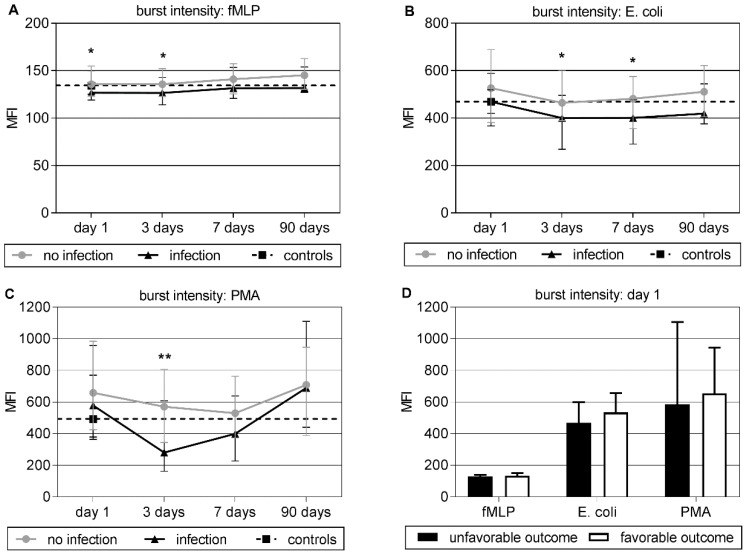
(**A**–**C**) Time course of neutrophil oxidative burst intensity, represented by the mean fluorescence intensity (MFI), under different stimuli with formyl-methionyl-leucyl-phenylalanine (fMLP), E. coli and phorbol 12-myristate 13-acetate (PMA) for patients without infection (dot), patients with infection (triangle), and controls (black square). Given are group medians and interquartile ranges. For better comparison, the control level is plotted as a dotted line over the entire time course. Significant results in day-by-day group comparison by Mann–Whitney U testing are marked as * for *p* < 0.05 and ** for *p* < 0.01. (**D**) Burst intensity under different stimuli at baseline (day 1) for the two outcome groups. There were no significant differences between groups.

**Figure 2:**
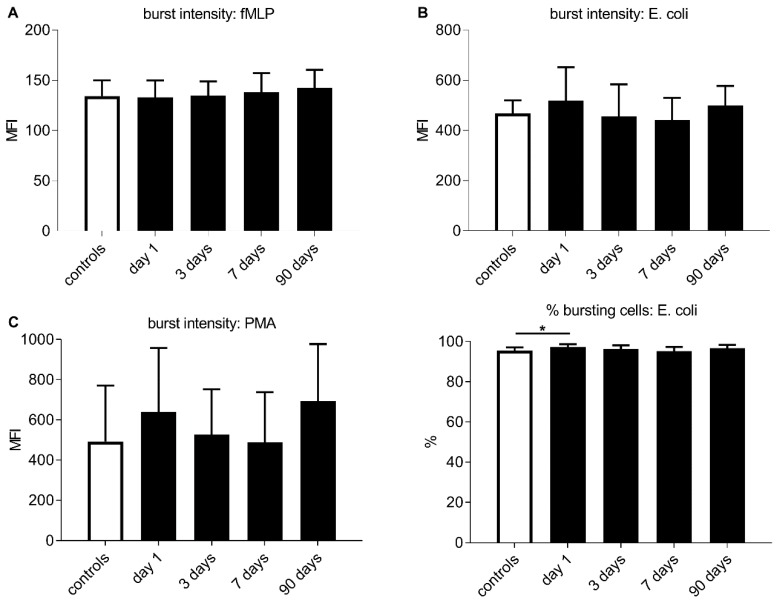
(**A**–**C**) Time course of neutrophil oxidative burst intensity represented by the mean fluorescence intensity (MFI) under different stimuli for all stroke patients (black bars) and control levels (white bars). (**D**) Time course of the percentage of bursting neutrophils after stimulation with *Escherichia coli* for all stroke patients (black bars) and control levels (white bars). (**A**–**D**) Given are group medians and upper interquartile ranges. Testing was performed using Mann–Whitney U for comparing burst intensity/percentage of bursting cells between all stroke patients and control levels. Significant results are marked as * for *p* < 0.05.

Baseline oxidative burst intensity as represented by the mean fluorescence intensity (MFI) was reduced in patients with infection compared to those without (median: 126.80, IQR: 119.00–135.90 vs. median: 135.70, IQR: 123.43–154.93; *p* = 0.013) using the weak stimulus fMLP. At day three, oxidative burst intensity was reduced in patients with post-stroke infections compared to those without for all stimuli and at day seven after stimulation with *Escherichia coli* (Figure 1A–C). Comparison of baseline oxidative burst intensity between all stroke patients and controls did not show any significant differences (Figure 2A–C).

#### 3.2.2. Percentage of Neutrophil Granulocytes Having Produced ROS 

The percentage of neutrophils showing oxidative bursts at baseline did not differ significantly between patients with infection and those without for any stimulus. The percentage of neutrophils showing oxidative bursts after stimulation with *E. coli* was moderately elevated in stroke patients compared to controls at baseline (*p* = 0.03, median: 97.30% vs. 95.51%) (Figure 2D). Eighteen controls (75%) showed levels of oxidative bursts after stimulation with *E. coli* below the estimated reference range of 97–100%.

### 3.3. Phagocytosis

#### 3.3.1. Phagocytic Capacity per Cell

**Figure 3:**
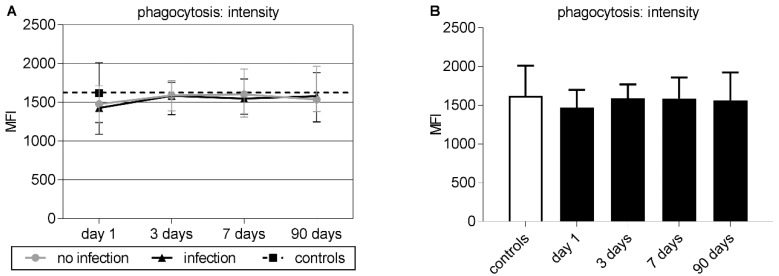
(**A**–**B**) Group medians and interquartile ranges of the phagocytosis intensity according to the incorporated amount of *E. coli* per neutrophil represented by the mean fluorescence intensity (MFI). (**A**) Time course of phagocytosis intensity for patients without infection (dot), patients with infection (triangle), and controls (black square). For better comparison, the control level is plotted as a dotted line over the entire time course. There were no significant results in day-by-day group comparison by Mann–Whitney U testing. (**B**) Time course for the phagocytosis intensity of neutrophils for all stroke patients (black bars) and control levels (white bars). There were no significant results between phagocytosis intensity of all stroke patients and control levels.

Baseline phagocytic capacity of neutrophils as represented by the MFI did not show a significant difference comparing patients with infection and those without (Figure 3A). Baseline phagocytic capacity was slightly but not significantly reduced in patients compared to controls (Figure 3B).

#### 3.3.2. Percentage of Neutrophil Granulocytes Having Phagocytosed Bacteria

The percentage of neutrophils that showed phagocytosis differed neither between patients with infection and those without nor between all stroke patients and controls (see Figure S1).

### 3.4. Neutrophil Phenotype

#### 3.4.1. CD11b

**Figure 4:**
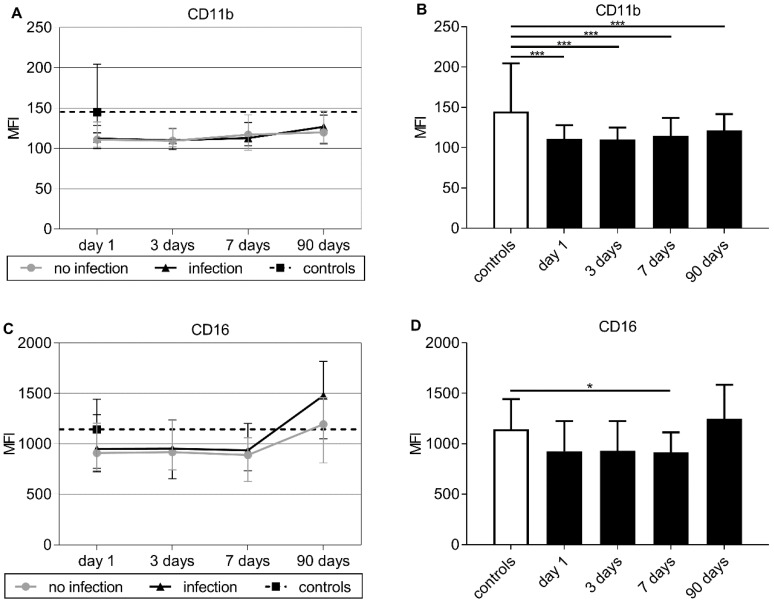
(**A**,**C**) Time course of CD11b and CD16 expression levels on neutrophils for patients without infection (dot), patients with infection (triangle), and controls (black square). For better comparison the control level is plotted as a dotted line over the entire time course. There were no significant results in day-by-day group comparison by Mann–Whitney U testing. (**B**,**D**) Time course of CD11b and CD16 expression on cell surface of neutrophils for all stroke patients (black bars) and control levels (white bars). Given are group medians and upper interquartile ranges. Testing was performed using Mann–Whitney U test for comparing receptor expression on neutrophils between all stroke patients and control levels. Significant results are marked as * for *p* < 0.05 and *** for *p* < 0.001.

The intensity of CD11b expression (MFI) did not significantly differ between patients with infection and those without at baseline nor at any other time point (Figure 4A). Compared to controls (median: 144.87, IQR: 128.71–204.54), the intensity of CD11b expression (MFI) at baseline was lower in stroke patients (median: 111.00, IQR: 101.30–127.90; *p* < 0.001). This difference persisted on days 3, 7, and 90 (*p* < 0.001 for each) (Figure 4B). The percentage of CD11b-positive cells was lower in all stroke patients compared to controls, but this difference did not reach statistical significance (see Figure S1).

#### 3.4.2. CD16

CD16 expression (MFI) at baseline was not different between patients with infection and those without (Figure 4C). CD16 expression in all stroke patients compared to control level tended to be reduced throughout the first week. This difference reached significance after seven days (median: 915.60, IQR: 699.70–1112.00 vs. median: 1142.04, IQR: 758.25–1442.25; *p* = 0.043) (Figure 4D). No relevant difference was seen for the percentage of CD16-positive cells—neither comparing patients with infection and those without nor comparing all stroke patients with controls (see Figure S1).

### 3.5. Independent Association of Oxidative Burst and Occurrence of Infections

Since fMLP-induced oxidative burst intensity (MFI), obesity, the infection-risk score (which includes i.a. NIHSS at baseline and S100B levels at day 3), and the NLR differed significantly between patient groups in the univariate analysis, we included these variables in a binary logistic regression analysis. The analysis revealed that the infection-risk score (*p* < 0.001; OR = 1.88, CI: 1.38–2.56) and fMLP burst intensity at baseline (*p* = 0.011; OR = 0.96, CI: 0.92–0.99) were independently associated with the development of infections after stroke.

### 3.6. Neutrophil Function and Clinical Outcome according to mRS and Barthel Index after 90 Days

Neutrophil function did not significantly differ between patients with favorable (*n* = 61) and unfavorable (*n* = 26) outcome concerning baseline oxidative burst intensity, percentage of neutrophils showing oxidative bursts, phagocytic capacity of neutrophils, the percentage of neutrophils showing phagocytosis, or the neutrophil phenotype (Figure 1D).

## 4. Discussion

The focus of our study was the association of neutrophil granulocytes’ function with the occurrence of infections in patients after acute ischemic stroke. As hypothesized, baseline neutrophil function, reflected by the production of intracellular reactive oxygen species (ROS) after stimulation with fMLP, was impaired in patients who developed an infection within the first seven days after stroke compared to patients with no infection. This association proved to be independent of known risk factors, and therefore might be an early sign of stroke-induced immunodepression. 

The oxidative burst intensity after treatment with stronger stimuli (PMA and *E. coli*) did not show a significant reduction in patients with infections before days three or seven after stroke. This could be explained by the stimuli’s different modes of activating oxidative burst generation. PMA causes intracellular activation of protein kinase C (PKC) and *E. coli* binds to Fc receptors and complement receptors from outside the cell, both initializing very effective intracellular pathways to induce oxidative bursts. In contrast, fMLP signals through G-protein-coupled receptors (GPCRs) induce a much weaker intracellular signaling pathway [20,21]. Thus, it could be possible that primarily only fMLP signaling is affected by immune alterations after stroke. 

Previous studies observed an impaired production of oxidative bursts after stimulation with fMLP in patients with ischemic and hemorrhagic stroke compared to healthy controls [5,9]. Impaired neutrophil oxidative bursts on admission to hospital have also been shown in patients with post-stroke infections, but without statistical significance. This lack of significance might be attributed to the small numbers in both of their groups (*n* = 8 and 14, respectively) [5].

Several studies reported an altered fMLP-signaling in other conditions such as cardiac surgeries or in response to exposure with bacterial extracts of *Klebsiella pneumoniae* [22,23]. 

A depressed superoxide production of neutrophil granulocytes after stimulation with fMLP was shown in apoptotic cells, whereas PMA-stimulated superoxide production was preserved [24]. In uremic and dialysis patients, impaired ROS production of neutrophil granulocytes after stimulation with fMLP (but not PMA) was observed, potentially explaining the high infection rates among these patients [25]. However, hemodialysis was an exclusion criterion in our study.

We could not confirm an impairment of neutrophil oxidative bursts in patients with acute ischemic stroke compared to controls, in general. The percentage of neutrophil granulocytes having produced ROS after stimulation with *E. coli*, however, was significantly lower in our control group compared to all stroke patients. According to literature, between 97% and 100% of neutrophil granulocytes produced ROS in healthy controls [5,26]. This is slightly different from our study where 18 (75%) of the controls were below this range (median 95.51%, IQR: 94.36%–97.09%). The cause for this difference remains unclear. 

Patients with post-stroke infections showed an elevated NLR on admission, which has recently been described as marker for stroke-induced pneumonia [19]. NLR has also been described before as marker for severe strokes [27], and might, therefore, indicate a higher vulnerability to post-stroke infections. In our cohort, more severe strokes (represented by higher levels of S100B and higher NIHSS) were found in the infection group. However, in multivariate analysis though, the NLR was no independent risk factor of post-stroke infections.

The expression of CD11b in stroke patients compared to that in controls was significantly reduced in our study. CD11b, a part of the complement receptor 3 (CR3)—also known as the MAC-1 receptor—is transferred to the cell surface upon activation of the neutrophil by proinflammatory stimuli [28,29]. It takes part in cell transmigration through the endothelium, recognizes and binds opsonized bacteria, and facilitates the internalization of pathogens [30]. It has been proposed that CD11b expression levels can be considered as a leucocyte activation marker [31]. It seems likely that reduced CD11b expression levels play some role in neutrophil dysfunction after stroke, although we could not show an association of CD11b expression and the occurrence of infections. Further investigations are needed to clarify the role of CD11b as part of CR3/MAC-1 receptor in post-stroke neutrophil dysfunction.

CD16 expression levels, as represented by MFI, also tended to be reduced in stroke patients compared to those in controls. CD16, also known as FcγRIII, is a cell membrane-bound receptor binding Immunoglobulin G (IgG). It regulates phagocytosis and, thereby, interacts with CD32 (FcγRII) inducing the engulfment of pathogens [32,33]. Reduced expression levels might contribute to a reduced phagocytosis of bacteria, which could not be proven by the results of our Phagotest^®^, though. Another explanation might be that CD16 is shed from the surface upon activation of the neutrophil in infection and inflammation, as described before [34]. Thus, reduced levels of CD16 could also reflect an activated functional status of the neutrophils regarding their phagocytic capacity.

In this study, baseline neutrophil granulocyte function and neurological outcome 90 days after acute ischemic stroke are evaluated. We assumed that a reduced neutrophil function is associated with an unfavorable outcome but did not find significant group differences in this respect. 

Strengths of our work include the thorough patient characterization and blood analyses in the hyperacute phase of stroke as well as rigorous follow-up examinations. However, there are some limitations that should be taken into account. The number of patients included into our study was too small to analyze a possible impact of factors other than stroke upon neutrophil function in stroke patients such as medication, comorbidities, infarct region, and stroke etiology, for example [35]. Considering the explorative character of the presented data and the multitude of factors that influence clinical outcome after acute ischemic stroke, the impact of neutrophil function must be re-assessed in a larger patient sample.

## 5. Conclusions

Impaired function of neutrophil granulocytes is associated with the development of post-stroke infections. While phagocytic function of neutrophils stayed unaltered after stroke, we found a link between reduced neutrophil oxidative burst activity in the hyperacute phase of stroke and the development of post-stroke infections. This association is independent from known clinical risk factors for the development of infections. Further studies will be needed to investigate whether neutrophil function may be used as biomarker to support risk assessment of post-stroke infections in a clinical setting.

## Figures and Tables

**Table 1:** Patient´s and control´s characteristics.

	Patients (*n* = 95)			Controls (*n* = 24)
	Infection = yes (*n* = 27)	Infection = no (*n* = 68)	*p*	
Female (%)	12 (44.44)	26 (38.24)	0.577	14 (58.33)
Age in years (IQR)	76 (68.00–84.00)	75.00 (64.00–82.00)	0.255	75.50 (68.00–79.00)
Arterial hypertension (%)	21 (77.78)	53 (77.94)	0.986	10 (41.67)
Smoking (%)	6 (22.22)	19 (27.94)	0.568	5 (20.83)
Hyperlipoproteinemia (%)	8 (29.63)	16 (23.53)	0.537	6 (25.00)
Diabetes mellitus (%)	2 (7.41)	13 (19.12)	0.158	3 (12.50)
Atrial fibrillation (%)	11 (40.74)	17 (25.00)	0.129	1 (4.17)
Obesity (BMI ≥ 30 kg/m^2^) (%)	8 (30.77)	7 (10.45)	0.015 *	3 (12.50)
Coronary heart disease (%)	6 (22.22)	16 (23.53)	0.892	2 (8.33)
Renal dysfunction (%)	5 (18.52)	17 (25.00)	0.499	n.a.
NIHSS on admission (IQR)	11 (7.00–17.00)	5 (3.00–7.75)	<0.001 ***	n.a.
S100B in µg/L on day 3 (IQR)	0.135 (0.096–1.410)	0.075 (0.050–0.116)	<0.001 ***	n.a.
Stroke subtype Cardioembolic (%) Large vessel (%) Small vessel (%) Other (%) Undetermined (%)	16 (59.26) 2 (7.41) 0 (0.00) 1 (3.70) 8 (29.63)	26 (38.24) 3 (4.41) 12 (17.65) 0 (0.00) 27 (39.71)	0.063 0.555 0.020 * 0.111 0.358	n.a.
Affected brain regions Anterior cerebral artery (ACA) (%) Middle cerebral artery (MCA) (%) Posterior cerebral artery (PCA) (%) Cerebellum (%) Brainstem (%)	5 (18.52) 25 (92.59) 2 (7.41) 2 (7.41) 0 (0.00)	4 (5.88) 53 (77.94) 8 (11.76) 6 (8.82) 5 (7.35)	0.058 0.093 0.533 0.823 0.148	n.a.
Mechanical thrombectomy (%)	4 (14.81)	6 (8.82)	0.391	n.a.
Intravenous Thrombolysis (%)	9 (33.33)	18 (26.47)	0.504	n.a.
CRP on admission (mg/L) (IQR)	3.30 (1.80–7.50)	3.10 (1.80–5.90)	0.454	1.30 (0.50–3.28)
WBC on admission (1000/µL) (IQR)	9.60 (8.10–11.80)	7.30 (6.28–9.28)	0.001 **	6.65 (5.68–8.05)
Neutrophil–lymphocyte ratio (NLR) on admission (IQR)	5.87 (3.21–9.31)	2.87 (2.00–4.36)	0.001 **	2.20 (1.66–3.29)
Infection-risk score (IQR)	3.00 (2.00–5.50)	1.00 (0.00–2.00)	<0.001 ***	n.a.
Unfavorable outcome	17 (68.00)	9 (14.52)	<0.001 ***	n.a.

Patient´s and control´s characteristics and clinical findings at day 1. Given are total numbers and percentages (%), respectively, medians and interquartile ranges (IQR) as well as *p*-values for the group comparison between patients with and without infection. Significant results are marked as * for *p* < 0.05, ** for *p* < 0.01, and *** for *p* < 0.001. NIHSS: National Institutes of Health Stroke Scale, mRS: modified Rankin Scale, BMI: body mass index, WBC: white blood cell count.

## Supplementary Materials

The following are available online at https://www.mdpi.com/2077-0383/9/3/872/s1, Figure S1: Time course for the percentage of bursting, phagocytosing, or receptor expressing neutrophils for all stroke patients (black bars) and control levels (white bars) (median and upper interquartile range), Table S1: Infection-risk score, Table S2: Patient recruitment.

## Abbreviations

BI	Barthel Index
BMI	Body mass index
CR	Complement receptor
CI	Confidence interval
CRP	C-reactive protein
DNA	Deoxyribonucleic acid
e.g.	Exempli gratia, for example
eGFR	Estimated glomerular filtration rate
FITC	Fluorescein isothiocyanate
fMLP	Formyl-methionyl-leucyl-phenylalanine
FSC	Forward scatter
FSC-H	Forward scatter-height
FSC-W	Forward scatter-width
GPCR	G-protein-coupled receptor
i.a.	Inter alia, among others
IQR	Interquartile range
MFI	Mean fluorescent intensity
MPO	Myeloperoxidase
mRS	Modified Rankin Scale
NET	Neutrophil extracellular traps
NLR	Neutrophil–lymphocyte ratio
NIHSS	National Institutes of Health Stroke Scale
PKC	Protein kinase C
PMA	Phorbol 12-myristate 13-acetate
ROS	Reactive oxygen species
SSC	Side scatter
TOAST	Trial of Org. in Acute Stroke Treatment

## Appendix A. Neutrophil Function Measurement

Reactive oxygen species (ROS) production and extent of phagocytosis by neutrophils was determined using the flow cytometry-based commercial test kits PHAGOBURST™ and PHAGOTEST™ (Glycotope Biotechnology) according to the manufacturer’s instructions with minor changes as follows: heparinized whole-blood samples were stored at room temperature for a maximum of four hours until work up was started. For oxidative burst measurement, 100 µL samples of whole blood were precooled on ice before adding either 20 µL of washing solution (negative control), formyl-methionyl-leucyl-phenylalanine (fMLP; weak stimulus), opsonized *E. coli* (strong stimulus) or phorbol 12-myristate 13-acetate (PMA; maximum stimulus) to stimulate generation of oxidative bursts. After 10 min in a water bath at 37 °C, 20 µL dihydrorhodamine 123 (which is converted to a fluorescent state in the presence of ROS) were added before further incubation for 10 min at 37 °C. Then, after erythrocyte lysis, a further centrifugation (5 min, 300 g, 20 °C) was performed. After a washing step, the remaining cells were incubated with 100 µL of fluorescent DNA-staining solution to avoid aggregation artefacts prior to measurement. Flow cytometry analysis was performed using BD FACS Canto™ II (BD Biosciences). For each sample, the neutrophil population was gated in the FSC-/SSC-Scatterplot and 10,000 events each were measured to assess the amount of ROS produced (i.e., oxidative burst intensity) as reflected by the mean fluorescent intensity (MFI) and the percentage of cells showing an oxidative burst.

To measure neutrophil phagocytotic capacity, a 100 L sample of precooled whole blood was incubated with 20 µL of fluorescein isothiocyanate (FITC)-labeled *E. coli* bacteria for 10 min in a water bath at 37 °C. Another 100 µL whole-blood sample remained on ice and served as negative control. After incubation, 100 µL of quenching solution was added into both samples to stop phagocytosis and minimize unspecific fluorescence of non-ingested bacteria. Erythrocyte lysis, DNA-staining, and measurement/gating were performed for both samples as described above. Percentage of neutrophils performing phagocytosis of FITC-labeled bacteria and the number of phagocytosed bacteria per cells (as reflected by MFI) were evaluated.
