# Supplementary material for: Early Post-Stroke Infections Are Associated with an Impaired Function of Neutrophil Granulocytes
Source: J Clin Med. 2020 Mar 23;9(3):872. doi: 10.3390/jcm9030872 (PMC7141520; doi:10.3390/jcm9030872)
Supplement: Supplementary file 1 [file jcm-09-00872-s001.pdf]

Supplemental Tables

Table S1. Infection-risk score.

Infection-risk factor	Scoring points
Reduced consciousness	1
Dysphagia	1
NIHSS > 8	1
High S100B (cutoff/median = 0.083 µg/L)	1
Urinary catheter	1
Invasive ventilation	1
Nasogastric feeding tube	1
Total:	7

Infection-risk score. S100B was determined at day three. NIHSS: National Institutes of Health Stroke Scale.

Table S2. Patient recruitment.

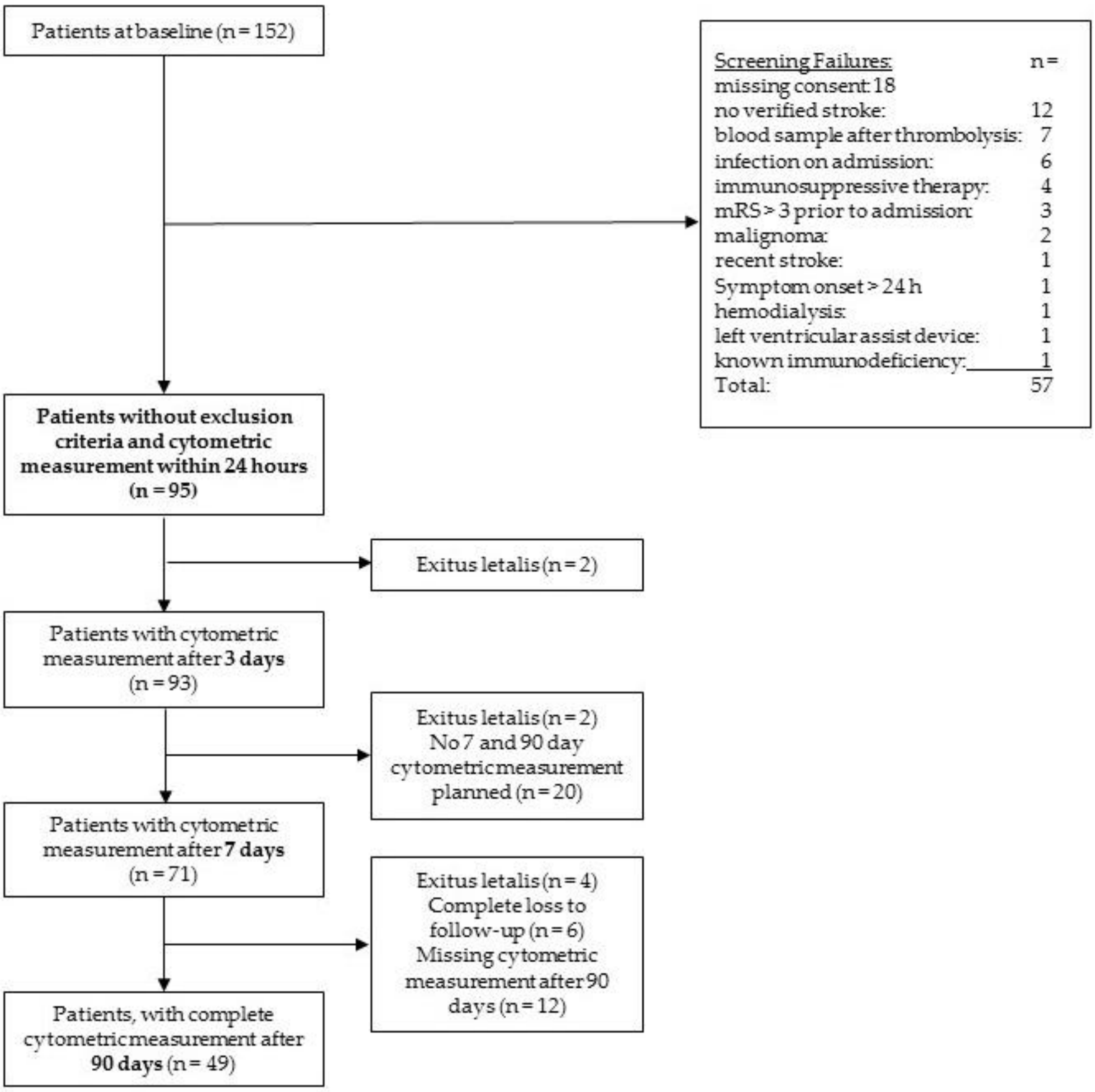

Left side: number of patients with determination of neutrophil function by cytometric measurement at the different follow-up time points (day 1, 3, 7, and 90). Middle: reasons for incomplete cytometric measurements. Right side: screening failures. As described above, 20 patients without infection did not receive the follow-up cytometric measurement of neutrophil function after 7 and 90 days, since

they did not develop infections. For 18 of those patients, clinical outcome could be assessed after 90 days. Eight patients died within the study period. mRS: modified Rankin Scale.

Supplemental Figure

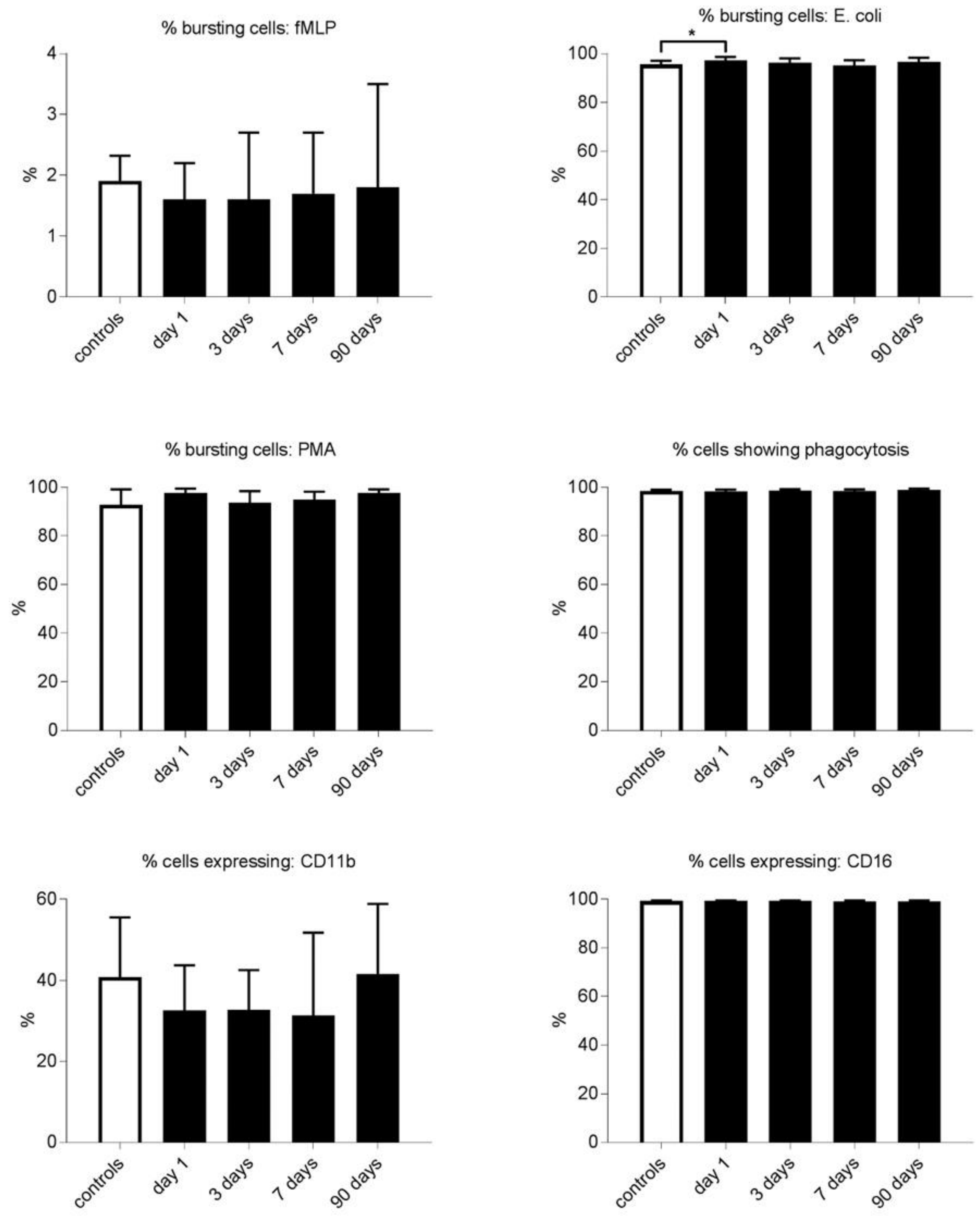

Figure S1. Time course for the percentage of bursting, phagocytosing, or receptor expressing neutrophils for all stroke patients (black bars) and control levels (white bars) (median and upper interquartile range). Testing was performed using Mann–Whitney U test for comparing percentages of cells between all stroke patients at day 1 and control levels. Significant results are marked as * for $p < 0.05$.
